# Spatial Requirements of Free-Ranging Huon Tree Kangaroos, *Dendrolagus matschiei* (Macropodidae), in Upper Montane Forest

**DOI:** 10.1371/journal.pone.0091870

**Published:** 2014-03-18

**Authors:** Gabriel Porolak, Lisa Dabek, Andrew K. Krockenberger

**Affiliations:** 1 Centre for Tropical Biodiversity and Climate Change, James Cook University, Cairns, Australia; 2 School of Marine and Tropical Biology, James Cook University, Cairns, Australia; 3 Department of Field Conservation, Woodland Park Zoo, Seattle, Washington, United States of America; The University of Wollongong, Australia

## Abstract

Tree kangaroos (Macropodidae, *Dendrolagus*) are some of Australasia's least known mammals. However, there is sufficient evidence of population decline and local extinctions that all New Guinea tree kangaroos are considered threatened. Understanding spatial requirements is important in conservation and management. Expectations from studies of Australian tree kangaroos and other rainforest macropodids suggest that tree kangaroos should have small discrete home ranges with the potential for high population densities, but there are no published estimates of spatial requirements of any New Guinea tree kangaroo species. Home ranges of 15 Huon tree kangaroos, *Dendrolagus matschiei*, were measured in upper montane forest on the Huon Peninsula, Papua New Guinea. The home range area was an average of 139.6±26.5 ha (100% MCP; n = 15) or 81.8±28.3 ha (90% harmonic mean; n = 15), and did not differ between males and females. Home ranges of *D. matschiei* were 40–100 times larger than those of Australian tree kangaroos or other rainforest macropods, possibly due to the impact of hunting reducing density, or low productivity of their high altitude habitat. Huon tree kangaroos had cores of activity within their range at 45% (20.9±4.1 ha) and 70% (36.6±7.5 ha) harmonic mean isopleths, with little overlap (4.8±2.9%; n = 15 pairs) between neighbouring females at the 45% isopleth, but, unlike the Australian species, extensive overlap between females (20.8±5.5%; n = 15 pairs) at the complete range (90% harmonic mean). Males overlapped each other and females to a greater extent than did pairs of females. From core areas and overlap, the density of female *D. matschiei* was one per 19.4 ha. Understanding the cause of this low density is crucial in gaining greater understanding of variations in density of tree kangaroos across the landscape. We consider the potential role of habitat fragmentation, productivity and hunting pressure in limiting tree kangaroo density in New Guinea rainforests.

## Introduction

The Huon tree kangaroo (*Dendrolagus matschiei*) is one of fourteen tree kangaroo species recognized by the IUCN, twelve species of which are endemic to New Guinea and two are endemic to Australia [1]. Huon tree kangaroos (*D. matschiei*) are endemic to high elevations of the Huon Peninsula, Morobe Province, Papua New Guinea, between 1,000 and 3,300 m above sea level, and a total geographic range of less than 14,000 km^2^ [2]. The Huon tree kangaroo is listed as Endangered [1]. Half of the fourteen species of *Dendrolagus* are considered to be endangered or critically endangered, threatened by hunting or habitat loss, with poorly understood ecology, small and restricted geographic ranges, and specialized diet and habitat requirements [1]. Tree kangaroos are an important component of New Guinea's endemic marsupial fauna with special significance for indigenous landowners [3] and consequently have an important role as conservation flagship species for motivating the public and decision-makers to ensure that Papua New Guinea's ecosystems are protected and well managed.

Despite being considered endangered, Huon tree kangaroos, along with New Guinea's eleven other tree kangaroo species, are poorly studied in contrast to the two species of tree kangaroo found in Australia [4] [5]. There is currently no information available on habitat requirements, home range or activity patterns of any New Guinean tree kangaroo species. Among other characteristics such as diet and predation, long-term conservation of Huon tree kangaroos (*D. matschiei*) requires better understanding of ecological characteristics such as home range size, potential seasonal shifts in range, core areas, and dispersal rates and patterns. This ecological knowledge combined with mapping techniques can be used to ensure that representative habitat and ecosystems are present within an existing or proposed protected area or management zones [6].

Range sizes and habitat use are better known in the two species of Australian tree kangaroos. Lumholtz's tree kangaroo, *D. lumholtzi*, which is restricted to the Atherton and Evelyn Tablelands of northeast Australia's wet tropical rainforests, has been the subject of studies of home range [7] [8], diet and behaviour [8] [9]. Lumholtz's tree kangaroos have small home ranges, ranging from 0.69 ha [7] to 2.1 ha [8] depending on the habitat type and amount of forest fragmentation. Female *D. lumholtzi* are relatively solitary and maintain discrete home ranges independent of other females, with only minor overlap at the margins [7] [8]. Newell [10] found that females occupied smaller ranges (0.69 ha) than males (1.95 ha), while the females in Coombes' [8] study had ranges as large as those of the males (2.1 ha average). Male *D. lumholtzi* maintained a home range independent of other males [7] [8] but have a greater tendency to overlap with adjacent males as well as with several females. Both studies of *D. lumholtzi* were conducted in highly fragmented forests. Bennett's tree kangaroos occupy a slightly larger home range than Lumholtz's tree kangaroo, (3.7–6.4 ha) [11]. Like *D. lumholtzi*, *D. bennettianus* generally have exclusive home ranges, but, while males remain solitary, females can share their home range with their offspring. It is unclear what factors drive the variation in tree kangaroo home range size, although the variation between Newell's [7] and Coombes' [8] measures may be related to the different habitat types in those studies, and Martin [11] has suggested that home range sizes of male *D. bennettianus* could be related to attributes of the individual males, such as body size, age and vigour rather than resources. Across other macropodid species, home range sizes are positively related to body size, but more strongly, inversely related to annual rainfall [12], with females of rainforest species occupying particularly small home ranges for their mass.

The spatial distribution reported by Newell [10], suggests that female *D. lumholtzi* may maintain ranges based on distribution of resources defended from other females whereas male spatial distribution is determined by the need to overlap several females [12]. Given the importance of female density in determining the reproductive rate of a population [13], spatial requirements of female tree kangaroos provide crucial information about potential population density and reproductive rate within a specific habitat.

This study describes the spatial use of habitat by Huon tree kangaroos (*D. matschiei*), focusing on estimating home range size as well as spatial distribution of male and females. Based on expectations from home range and spatial distribution of Australian tree kangaroos and other rainforest macropodids [7] [12], we expect female Huon tree kangaroos to have smaller, discrete home ranges with little overlap between adjacent individuals while males may have larger ranges overlapping with several females. This type of spatial arrangement would make it possible to estimate the density of tree kangaroo populations and support the development of effective management strategies to conserve populations of Huon and other tree kangaroos in the wild.

## Materials and Methods

This study was conducted from 2004–2007 in upper montane forest at a locality known as Wasaunon in the Sarawaget Ranges on the north coast of the Huon Peninsula, Papua New Guinea (146°54′52.90″ East; 6°5′31.68″ South). The study site is located approximately 9 km from the nearest village in continuous primary forest, which was hunted up until 20 years prior to its protection in 2002. It is above the elevation that people cultivate in this landscape and further than the forests where people harvest building materials and consequently shows little evidence of broad-scale anthropogenic influences.

The data are lodged with the James Cook University Tropical Data Hub (https://research.jcu.edu.au/researchdata/default/detail/ef04f467900305d8dd8755715067cd6a/). The study area is about 984 ha, but contiguous and within a large tract (∼60,000 ha) of relatively undisturbed forest in the YUS Conservation Area [14]. Wasaunon is at an altitude of 3000 m above sea level, with an average rainfall of approximately 2500 mm *p.a.*, average minimum temperature of 5°C and annual average maximum temperature of 30°C. Rain occurs throughout the year, although the wettest season occurs from November through March and the driest season from June through September. The site supports an upper montane forest dominated by *Dacrydium*, *Decaspermum*, *Syzygium*, and *Dicksonia* tree species [15] with an average canopy height of 28–30 m.

### Ethics Statement

This study was conducted in accordance with Papua New Guinea law with approval of the PNG National Research Institute and permission of the relevant indigenous landowners. Animal care and handling techniques complied with the Australian National Health and Medical Research Council's Code of Practice for Care and Use of Animals for Scientific Purposes (2004) and was approved by the James Cook University Animal Ethics Committee (A590, A1928).

Huon tree kangaroos (*D. matschiei*) were located for the study by a team of 6–8 local landowner hunters searching visually within the vicinity of one kilometre of the camp. After sighting a tree kangaroo, the hunters used a traditional method to live-capture the animal. The undergrowth within a radius of approximately 10 m around the tree in which the tree kangaroo was sitting was rapidly cleared and the cut vegetation was piled around the perimeter to create a temporary barrier, known in the local language as an “*im*”. One hunter then climbed a neighbouring tree and encouraged the tree kangaroo to jump to the ground, where it was hand-captured by the base of the tail, within the “*im*”. The captured tree kangaroo was then quickly placed into a hessian bag, which helped to minimise stress on the animal while it was transported back to the camp. The capture process took approximately 15–20 minutes once the animal had been sighted and generally occurred in the early hours of the day (0800 – 1200).

Each tree kangaroo was handled under the care of a field veterinarian and routinely sedated for measurements and handling, either by inhalation of anaesthetic (Isoflurane: Oxygen 0.5%–1.5% to effect; Halocarbon Products corporation, New Jersey, USA) or injected sedative (Telazol: *I.M.* 2 mg/kg; Fort Dodge Animal Health, Iowa, USA). Animals were then weighed, measured (body length, tail length, head width/length), and fitted with a radio transmitter mounted on a collar (MOD-205 VHF Transmitter; Telonics Incorporation, USA). Animals were marked with PIT tags (AVID Microchip Company, CA, USA) implanted subcutaneously and suprascapularly. They were then kept under observation for a period of at least four hours. When they had sufficiently recovered they were released at the point of capture.

The radio-collared tree kangaroos were located daily for six months using a hand-held radio receiver (AVM – LA12-Q receiver, AVM Instrument Company, CA, USA) with a three-element Yagi antenna. Locations were confirmed visually where possible (54% of locations were confirmed visually) and the position recorded using GPS (Garmin 12CX, Garmin International Inc, KS, USA or GeoExplorer® 3, Trimble Navigation Ltd CA, USA).

The home range area for each individual was calculated according to three different methods: Harmonic mean (HM), Kernel (KM) and Minimum Convex Polygon (MCP), using Ranges6 software [16]. The probabilistic methods (HM, KM) were included to provide information about the distribution of activity within the ranges (i.e. cores) and the MCP method was included to provide comparisons with other studies. The number of locations required to adequately define home range were determined by the incremental area analysis function of Ranges6 and showed that at least 70 locations were needed in this study. Three individuals with less than 70 locations were discarded from further analysis.

Grid size was estimated through visual analysis of contour plots that showed minimal cluster between individual contours. In this case the default (40 m×40 m) cell size in Ranges6 was the most appropriate for this study/analysis. The smoothing factor is a variable that modulates the density estimated by a kernel function to vary the tightness with which contours conform to locations [17]. This variable was determined by identifying a point in the kernel analysis where contours showed conformity towards the locations (smoothing factor = 40 in this study).

**Figure 1:**
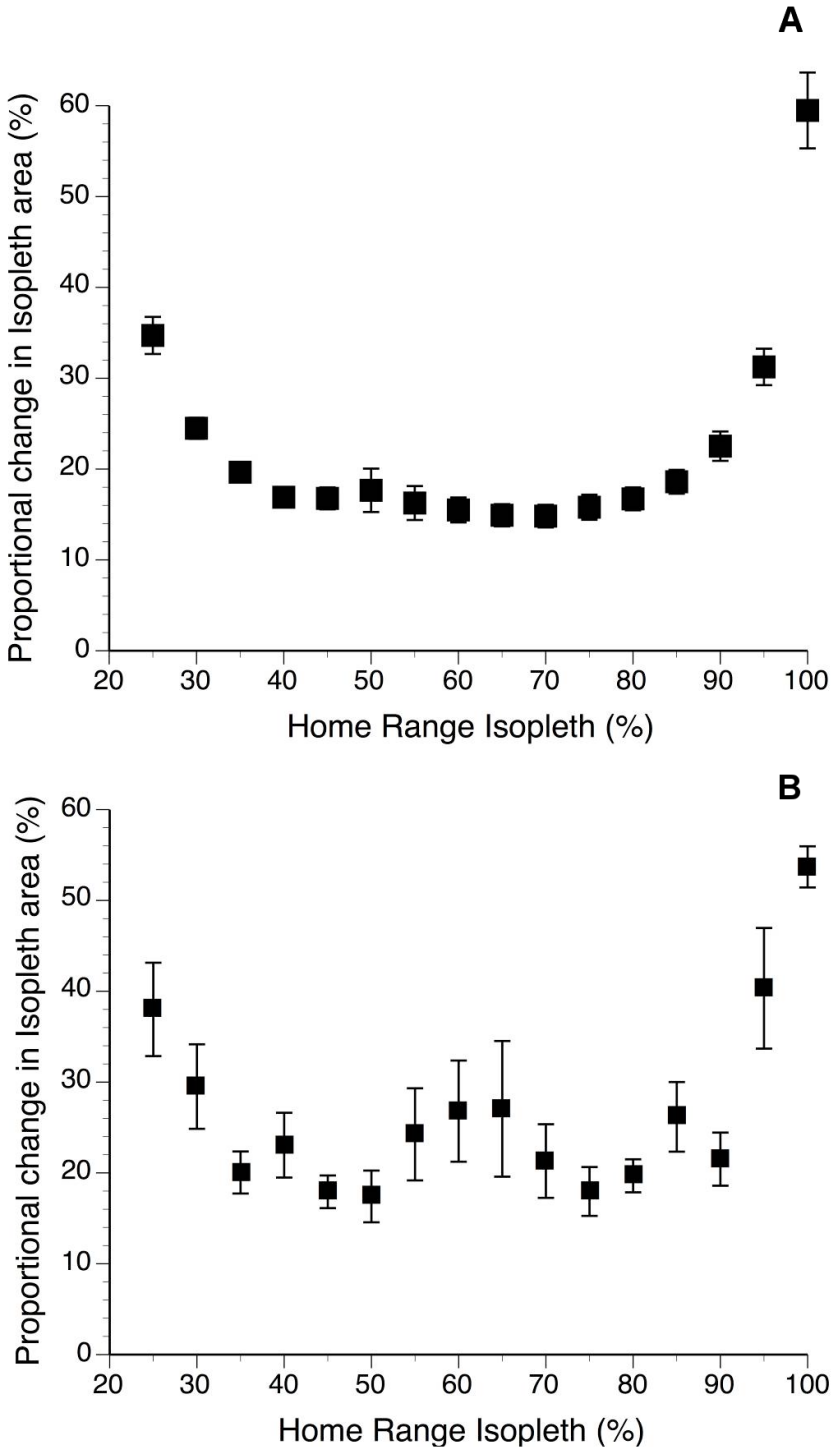
The proportional incremental change in home range area, moving out from the centre of activity in increments of 5% isopleths (means ± standard error; n = 15). Core areas corresponded to minima on the curve and the 90% isopleth was taken to represent the entire range- excluding the strong effects of outliers that increased the incremental changes at more inclusive isopleths (i.e. 95% and 100%). A) Harmonic mean; minima are at 45% and 70%. B) Kernel; minima are at 50% and 75%.

Home range cores were determined at the isopleths where the incremental change in home range size was minimized. The Harmonic mean cores defined in this way were at 45% and 70% (Fig. 1a), while the Kernel cores were at 50% and 75% (Fig. 1b). In both cases the 90% isopleths were used to define the entire range because it avoided undue emphasis on outliers that caused rapid increase in incremental change of area at isopleths above 90% (Fig. 1) [17]. These isopleths have been used to define the home ranges in the results. Results for the 95% isopleths have also been included, as well as 50% isopleths HM and 70% isopleth KM results, for comparison with other studies which commonly report 50, 70 and 95% isopleths.

Overlap between home ranges of neighbouring individuals was calculated using the Ranges6 software, at core area isopleths of 45% and 90% for harmonic mean only. We chose to report overlaps between individuals only with respect to the harmonic mean ranges, because that is the most common method used in the literature, but the patterns of kernel ranges were similar. To avoid comparisons of animals that were not neighbours, overlap was only calculated for pairs of individuals that had some overlap at the 90% harmonic mean isopleth. Home range sizes of males and females were compared using Student's t-test [18] with adjustment for heterogeneous variances when required.

## Results

Field observations showed that in 90% of 1,534 daily locations, Huon tree kangaroos were in the canopy at an average height of 18–20 m high, while the remaining 10% of locations were on the ground.

Huon tree kangaroos (*D. matschiei*) had large home ranges, averaging 81.3±16.9 ha (SEM, n = 15, 90% HM isopleths; Table 1), that overlapped extensively (90% HM isopleths; 20–34%) with their neighbours (Table 2). There was no statistical difference between the home range size of males and females at any core of any of the three calculation algorithms used in this study.

**Table 1:** Home range areas (ha) for adult male and female Huon tree kangaroos (*D. matschiei*) in upper montane forest at Wasaunon on Papua New Guinea's Huon Peninsula (means ± SEM).

	Mass (kg)	Algorithm	45%	50%	70%	75%	90%	95%
**Males**	6.8±0.3	**HM** 1	21.7±7.0	25±8.1	38.6±13.1	50.5±17.6	81.8±28.3	103.2±35.1
(*n* = 7)		**Kernel** 2	13.5±4.6	16.1±5.8	27.6±9.5	40.1±13.8	72.4±24.7	99±32.3
		**MCP** 3						120.4±38.6
**Females**	7.5±0.2	**HM**	20.4±5.1	23.4±5.9	34.7±8.9	46.9±11.8	80.8±20.3	108.7±27.5
(*n* = 8)		**Kernel**	10.2±1.7	11.8±2.0	24.5±6.8	33.9±9.1	65.5±17.2	95.9±28.0
		**MCP**						156.5±37.6
**Mean**	7.2±0.2	**HM**	20.9±4.1	24.2±4.8	36.6±7.5	48.6±9.9	81.3±16.5	106.2±21.2
(*n* = 15)		**Kernel**	11.7±2.3	13.8±2.9	25.9±5.5	36.8±7.8	68.7±14.2	97.4±20.5
		**MCP**						139.6±26.5

1 Harmonic mean algorithm.

2 Kernel mean algorithm.

3 Minimum convex polygon algorithm (100%).

**Table 2:** Proportion of home range area overlap between adjacent Huon tree kangaroos (*D. matschiei*) in upper montane forest at Wasaunon on Papua New Guinea's Huon Peninsula (mean ± SEM).

	Proportion of overlap (%)	
	45% HM	90% HM
Females (*n* = 15)	4.84±2.93	20.79±5.48
Males (*n* = 10)	12.32±6.34	34.00±7.20
Males & females (*n* = 30)	13.47±3.62	34.43±4.46

**Figure 2:**
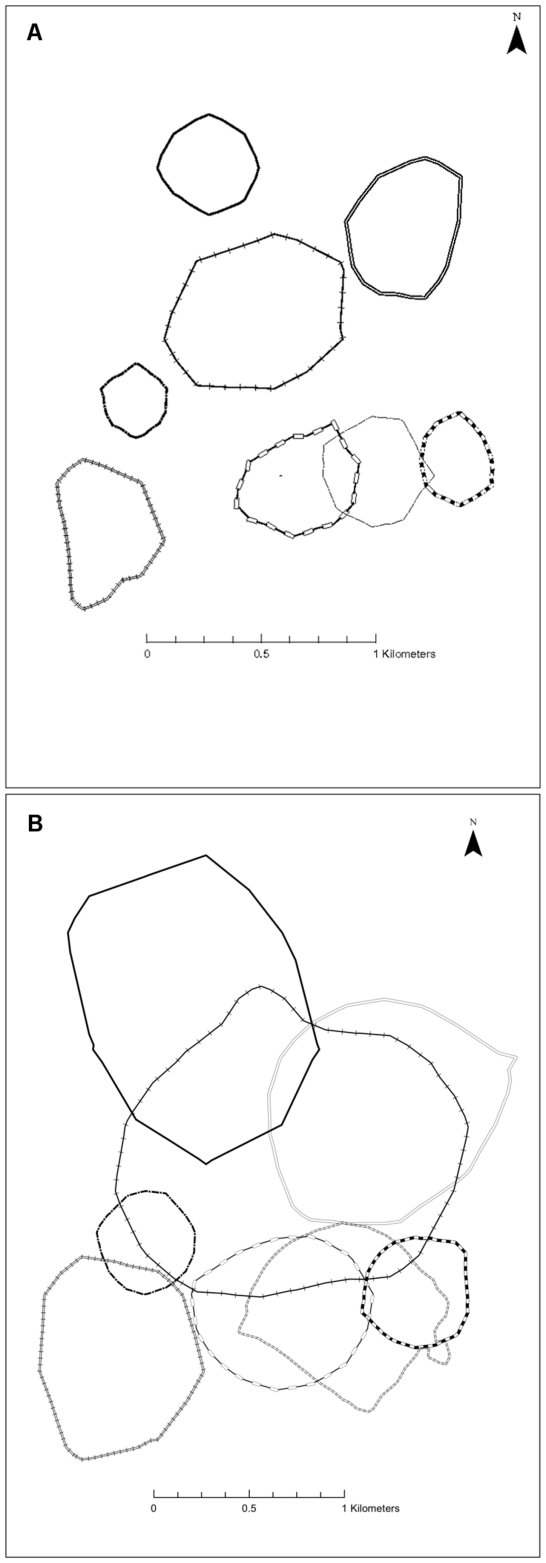
Home ranges of female Huon tree kangaroos, *D. matschiei*, at Wasaunon, Huon Peninsula, Papua New Guinea. A) 45% harmonic mean isopleth core areas with minimal overlap between neighbouring females. B) 90% harmonic mean isopleth areas with extensive overlap between neighbouring females. Ranges of individual females are denoted with different line styles.

**Figure 3:**
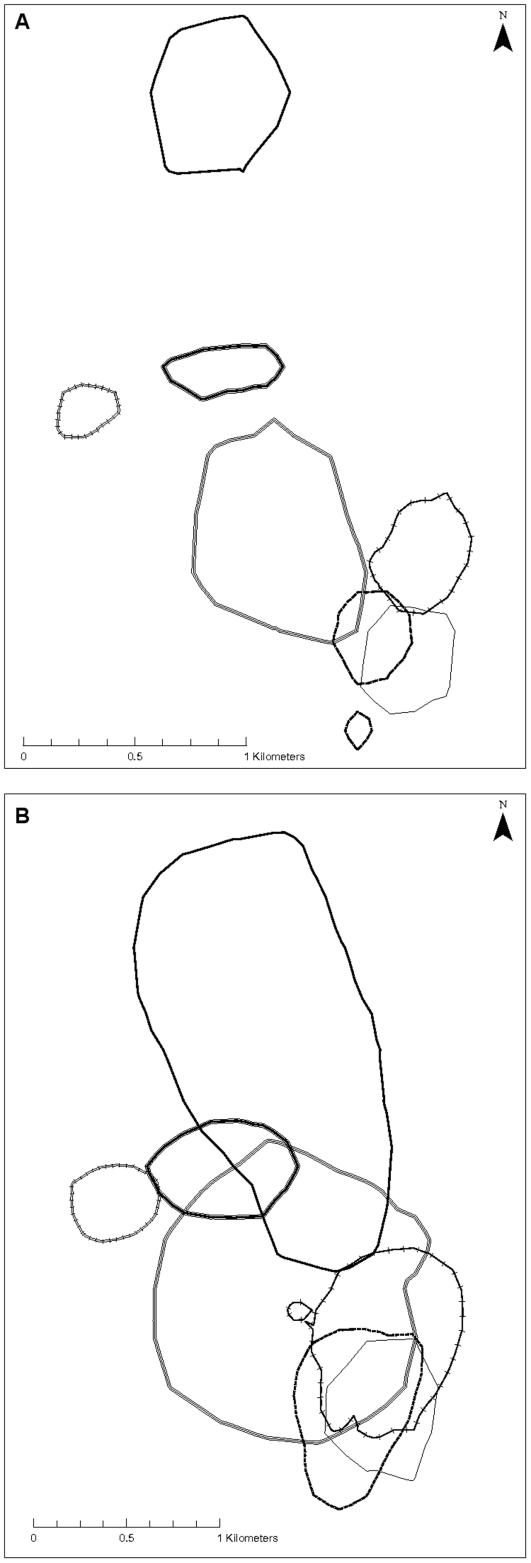
Home ranges of male Huon tree kangaroos, *D. matschiei*, at Wasaunon, Huon Peninsula, Papua New Guinea. A) 45% harmonic mean isopleth core areas with minimal overlap between neighbouring males. B) 90% harmonic mean isopleth areas with extensive overlap between neighbouring males. Ranges of individual males are denoted with different line styles.

**Figure 4:**
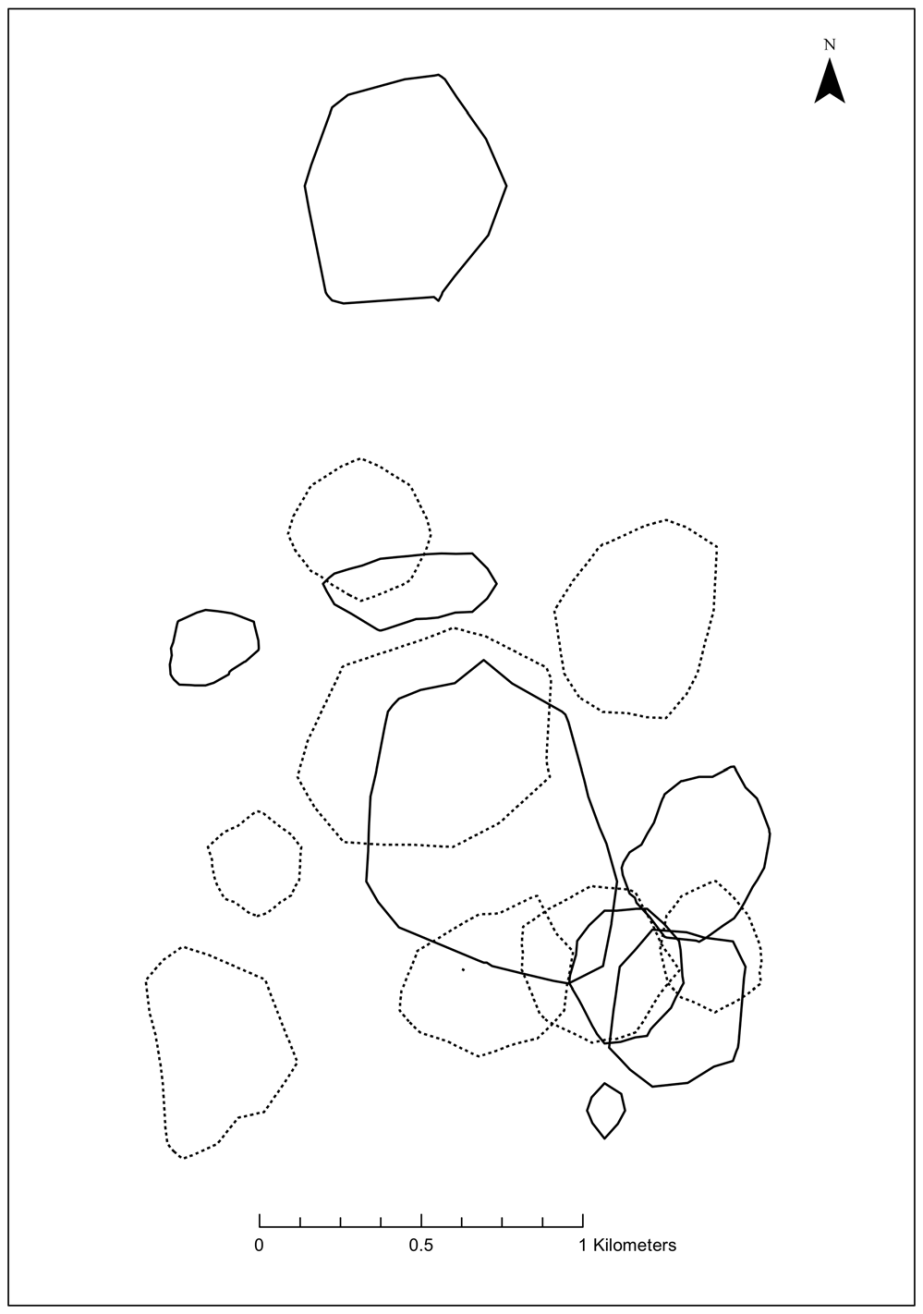
Spatial arrangement of the genders in Huon tree kangaroo home ranges. These are 45% harmonic mean isopleth core areas, showing that both males and females have relatively exclusive core ranges with respect to their own gender, but that males tend to overlap several females. Female ranges are denoted by broken lines and males by unbroken lines.

Huon tree kangaroos had cores of activity within their range at 45% (20.9±4.1 ha) and 70% (36.6±7.5 ha) harmonic mean isopleths (Fig. 1a). There were similar cores within the Kernel mean calculated ranges, at the 50% and 75% isopleths (Fig. 1 b). Despite the extensive overlap at the level of the entire range (90% isopleths), at the level of the smaller core (45% HM) there was little (4.8±2.9%) overlap between adjacent females (Fig. 2a; Table 2), but slightly more between adjacent males and between males and females (Fig. 3; Table 2). Consequently, at the core (45% HM) female Huon tree kangaroos had relatively exclusive ranges (Fig. 2a), overlapped by male Huon tree kangaroos that each tended to overlap several females (Fig. 4).

## Discussion

This study provides the first information on the movements and home range size of any New Guinean tree kangaroo species, substantially expanding our understanding that has previously been restricted to a few studies of Australian tree kangaroos [7] [8] [9] [11]. The tree kangaroos in this study had the largest home range size recorded for any tree kangaroo species (81.8±28.3 ha; 90% HM), and larger core areas of activity (45% (20.9±4.1 ha) and 70% (36.6±7.5 ha)), which was between 40 and 100 times larger than ranges measured for the similar sized Lumholtz's tree kangaroo (Table 1) [7] [8], higher than any other rainforest macropodid and closer to xeric-adapted species such as *Macropus dorsalis* [19]. Male and female Huon tree kangaroos also ranged over similar areas, in contrast with Newell's [7] study of *D. lumholtzi*, where males had substantially larger ranges than females. Understanding this large variation in home range between tree kangaroo species is particularly important to understanding the space use and habitat requirements for conservation of tree kangaroos. In this study we have reported results using a variety of calculation techniques (Harmonic mean, Kernel and Minimum Convex Polygon) to maximize the potential for comparability with past and future studies. However, given that the pattern of results is very similar between the harmonic mean and Kernel techniques, we only discuss the results of the harmonic mean algorithm, as it is the most commonly used technique in the literature.

There are three effects that may explain the large variation between the home range of the Huon tree kangaroo and its Australian congeners: habitat fragmentation effects, altitude and its effects on productivity, and effects of past hunting. Habitat fragmentation is widely regarded as a major threat to the persistence of wildlife populations [20] [21] [22], including tree kangaroos [10]. However, little is known about mechanisms underlying population responses to fragmentation [23] [24] [25]. The studies of *D. lumholtzi* were conducted in strongly fragmented habitat, whereas this study was conducted in largely intact primary rainforest. Clearing of forest vegetation for agriculture or settlements results in a reduction of available habitat and, more particularly, in the fragmentation of habitat [26]. Habitat fragmentation determines the distribution of resources in the environment [25], which in turn largely determines spatial distribution of individuals within it [27] [28]. For example, brush-tailed phascogales (*Phascogale tapoatafa*) and squirrel gliders (*Petaurus norfolcensis*) in fragmented roadside habitats had substantially smaller home ranges than individuals in continuous forest [29] [30], possibly associated with a higher density of large trees and higher habitat quality in small fragmented areas. The roadside was protected within an agricultural landscape of relatively high nutrient soils [29], whereas the continuous forest had not initially been cleared, as it was less suitable for agriculture. The authors [29] [30] interpreted the smaller range size of females in the fragmented habitat as indicating higher habitat quality of these fragments because habitat quality and environmental productivity are major determinants of home range size [31] and female home ranges reflect resource availability [32] [33]. Habitat fragmentation can also alter social distributions. The distribution of male and female mammals within a habitat affects the mating patterns of populations [32] [34]. Therefore, habitat fragmentation has the potential to influence the social and mating systems of a population [35] by influencing the spatial distribution of individuals [36]. However, given that all other studies of rainforest macropodids show small home range sizes [12], not just *D. lumholtzi*, it seems unlikely that the contrast between *D. lumholtzi* and *D. matschei* is a result of fragmentation reducing range sizes of *D. lumholtzi*, but also that *D. matschiei* in this study had larger home ranges.

Secondly, the large home range size of the Huon tree kangaroo may be due to effects of elevation on habitat productivity and plant diversity. Plant species-richness and diversity decreases with elevation [37] and the accompanying decrease in average temperature slows plant growth [38]. This could result in lower productivity of the high elevation (3000 m) Huon tree kangaroo habitat in this study compared to studies of the Lumholtz's tree kangaroo conducted at 700 m elevation. If we assume that an animal of energetic requirements *R* (kJ/day) utilizes the minimum area that can sustain its energetic requirements and the environment provides utilizable energy for that specific trophic niche at a rate *P* (kJ/day/unit area). Home range (*H*) thus becomes *H* = *R/P* [39]. Thus animals in habitats of high productivity will have a smaller home range than animals in habitats of lower productivity. Conversely, an animal living in a habitat of low productivity should have a larger home range than that predicted by the generalized positive relationship between home range and body weight [40]. Consequently, at the broad scale home range size is related to variables such as latitude and precipitation [12] [41], mediated through productivity [39] [42]. This is confirmed by experimental studies of a range of mammals showing a negative relationship between food availability and home range size [33] [43] [44] [45] [46]. As increasing altitude is associated with decreasing primary productivity, we would expect home range size of species within a given trophic niche to increase with altitude. Thus, low productivity of high altitude habitat may force the Huon tree kangaroo to maintain large home ranges to include sufficient resources for maintenance and reproduction. The limited observations we have of *D. matschiei* suggest that diet is similar to that described for other tree kangaroos, with possibly a lower use of mature foliage (pers. obs.).

Lastly, the current study was conducted in an area that has had reduced hunting over the past 20 years due to adoption of Seventh Day Adventist practices that proscribe consumption of bushmeat. In addition, in 2002 this area was protected for the YUS Conservation Area. However, hunting is an important customary practice in Papua New Guinea [47] [48], and the effects over many years of past hunting have influenced population distribution. In comparison, hunting has not been an important influence on tree kangaroos in Australia for a much longer period [4]. Hunting of wildlife for human consumption has been identified as both a conservation and human livelihood issue [49] because it can lead to a decline in population of the target species [49] [50] [51]. Hunting is especially problematic in the humid tropics, where the low biological production of large bodied animals frequently cannot meet the hunting pressure [52]. Hunting could have direct and indirect effects on density and range size of tree kangaroos. For example, hunting could have reduced the density of *D. matschiei* below what the carrying capacity of the habitat could have been, without hunting pressure. This low density might allow individuals to maintain larger home ranges because of low numbers of interactions with their neighbours, leading to a dynamic adjustment between reduced densities and increased range size. Mammals frequently tolerate large amounts of overlap in the areas they use [53] [54] [55] as well as the peripheral area of their home range, territories and core areas [56]. In this inferred scenario the low density of *D. matschiei* in this study would have low numbers of territorial encounters with their neighbours and so are tolerant of overlap, whereas the high density populations of *D. lumholtzi* studied by Newell [7] and Coombes [8] would have large numbers of interactions with their neighbours that promote more intense territorial defence and thus not only smaller ranges, but also lower tolerance of overlap. Consequently, if this pattern is consistent, and either altitude or hunting pressure has contributed to the large ranges seen in this study, then we might expect that either in lower altitude habitat, or with recovery of population after cessation of hunting, the pattern of smaller, but exclusive ranges seen in *D. lumholtzi* would apply also to *D. matschiei*.

Hunting can also directly affect the behaviour of prey animals, influencing them to maintain lower densities to avoid predators and hunters [3]. Martin [57] suggests that Bennett's tree kangaroos were once restricted to “*taboo*” sites (Mt Finnigan) located on traditional Aboriginal land on Shipton's Flat in far northeast Queensland. This was attributed to no-hunting practices on sacred land where Aboriginals believed their ancestors originated. Traditional hunting has decreased over the past few decades and Bennett's tree kangaroos are now commonly found in the lowlands outside those “taboo” sites.

Unlike *D. lumholtzi*, whose females are effectively solitary and maintain exclusive ranges with little overlap from neighbouring females at the 90% HM isopleths [7], ranges of female *D. matschiei* overlap extensively with their neighbours (Table 2; Figure 2b). The 90% HM isopleths provided a good estimation of the total area utilised by an individual by encompassing all rarely used outlying locations (Figure 1a) [17]. However, female *D. matschiei* do maintain a core (45% HM, 50% KM) within their range that is close to exclusive (Table 2; Figure 2a). Identifying the core area provides an important theoretical framework for describing selected areas that contain resting sites, shelter, and reliable food sources for these tree kangaroos [58]. In this study, we used a numerical procedure to determine core areas that made no assumptions about the likely cores, but rather defined cores as the isopleths where the incremental increase in range size was minimized. The core areas we describe were defined by the way that individual tree kangaroos structured their activity within their range, as relative concentrations of activity; and, therefore, have greater ecological significance compared to studies that use an *a priori* definition, and commonly define the “core” as either 50% or 70% isopleths [17] [59]. Within the core of activity, males overlapped more with females and other males than did pairs of females, which is consistent with the other polygynous species and with *D. lumholtzi*. The approach used in this study to define the core home range was similar to that used by Coombes [8] who also found similar exclusive core areas at 55% and 75% HM for both males and females, in contrast to this study where male core areas overlapped with several females on a ratio of 1∶3 (males: female). In Newell's [7] study, female ranges were exclusive (90% HM), but males overlapped several females. We suggest that the pattern of male and female ranges in *D. matschiei* is broadly similar to that in *D. lumholtzi* [7] [8], and that female ranges are likely to be determined by the need to encompass sufficient resources, whereas male ranges are also determined by the need to overlap the ranges of several females [12].

Apart from providing insight to the mating system, the identification of core area is useful in the estimation of population density in mammals *i.e.* how much space each animal requires in that particular habitat [32] [42] [60]. Alternatively, core areas can also identify resource availability, because home range size and resource abundance have an inverse relationship [42]. Either way, female density is particularly important in conservation biology because females determine the reproductive rate of the population [13]. From the exclusive core area of 19.4 ha (20.4 at 45% HM and 4.8% overlap), we can provide the first estimate of density for *D. matschiei*, which is one female per 19.4 hectares in this habitat. We have not used this density to estimate the local population of tree kangaroos because it is based on assumptions that are too weak. Therefore we cannot yet estimate the population number of *D. matschiei* throughout its range or in the YUS Conservation Area. A simple extrapolation of this sort assumes that all the land pledged for conservation is suitable tree kangaroo habitat, and the density equal across that area. If the carrying capacity of the habitat for tree kangaroos is strongly affected by productivity, driven by an altitudinal gradient of temperature, as discussed above, much of the pledged area is at lower altitude and could have higher densities of tree kangaroos. If, on the other hand, the density of tree kangaroos at the Wasaunon study site was depressed by the impacts of past hunting, as discussed above, then much of the pledged area is closer to villages and likely to have sustained higher hunting pressure in the past, with consequent lower density [3]. Clearly, we still need to understand the variation in quality of the habitat and consequent variation in density of tree kangaroos across the landscape in order to provide better population estimates.

Neighbouring tree kangaroos overlapped each other extensively at the level of the entire range (90% HM; Table 2; Figure 2b & Figure 3b). This is important because it clearly signifies that in this study, *D. matschiei* did not have exclusive home ranges, outside the inner cores. This finding differs from studies conducted on the Australian Lumholtz's tree kangaroo, which show that females have exclusive home ranges, while males overlap with other males as well as with several other females (90% HM) [7]. This apparent tolerance of overlap with adjacent females could be associated with small dispersal distances by females that would lead to high degrees of relatedness between adjacent females [61], so the tolerated neighbours may be sisters or mother and daughter, as proposed by Coombes [8] for an overlapping pair of females in her study. The two tree kangaroo species may be equally solitary, but range size and overlap may interact in a complex way with density as described above.

The assessment of population density is a key issue in ecology and conservation biology. Experimental studies have shown population density and habitat area to be strong predictors of extinction and vulnerability [62] [63]. This study provides a reference point for population density and range size that can be used in assessing the value of specific management actions. Radio telemetry studies can facilitate management actions by identifying suitable habitats and ensuring these areas are large enough to support wildlife populations. The availability of resources to conservation programs is limited and data on endangered species are often inadequate or unavailable, yet scientifically reliable estimates of minimum viable population (MVP) sizes and habitat areas are essential [64] for effective conservation decision making. This study has contributed to the study of *D. matschiei* by providing the first estimates of population density and habitat area required by a New Guinea tree kangaroo species as part of a broader habitat conservation program. We have also identified potential mechanisms underlying variations in the density of tree kangaroos. The predicted effects of those mechanisms, changes in range size and density over altitudinal gradient or over time in response to cessation of habitat fragmentation and hunting, can be experimentally tested and used in developing adaptive management strategies for this species. Quantifying the variations in home range size, density and relative abundance at a broader scale across the landscape would be a valuable addition to our knowledge of Huon tree kangaroos, allowing more robust estimation of populations and their spatial requirements. Furthermore, quantification of the impacts of hunting in this landscape would provide a realistic assessment of the threat to populations. Coupled with population estimates this would allow estimation of population viability and the role of the newly established protected area. Dissemination of this information to local communities will reinforce the long-term benefits of conservation for sustainable use of their forest resources.
